# Effects of natural environmental conditions on faecal glucocorticoid metabolite concentrations in jaguars (*Panthera onca*) in Belize

**DOI:** 10.1093/conphys/cou039

**Published:** 2014-09-09

**Authors:** J. Bernardo Mesa-Cruz, Janine L. Brown, Marcella J. Kelly

**Affiliations:** 1 Department of Fish and Wildlife Conservation, Virginia Polytechnic and State University, Cheatham Hall, 310 West Campus Drive, Blacksburg, VA 24061, USA; 2 Center for Species Survival, Smithsonian Conservation Biology Institute, 1500 Remount Road, Front Royal, VA 22630, USA

**Keywords:** Faecal glucocorticoid metabolites, hormone degradation, immunoassay validation, jaguar, non-invasive monitoring, *Panthera onca*

## Abstract

*In situ* studies that rely on non-invasive faecal hormone monitoring are subject to problems due to potential changes in hormone concentrations in samples exposed to field conditions. In this study, we conducted an environmental validation for measurement of faecal glucocorticoid metabolites (FGMs) in jaguars (*Panthera onca*). We collected fresh faeces (e.g. no older than 8 h) from jaguars (six males and four females), housed at the Belize Zoo, and exposed them randomly to two environmental conditions: shade and sun. A control (first sub-sample) was immediately frozen, after which sub-samples were frozen daily over a 5 day period in both the dry and wet seasons. We quantified FGMs using a cortisol enzyme immunoassay (EIA) and a corticosterone radioimmunoassay (RIA), both capable of identifying relevant metabolites. Results indicated that FGMs assessed with the cortisol EIA were stable for 5 days during the dry season but for <1 day during the wet season, while FGMs assessed with the corticosterone RIA were stable for 5 days during both the dry and wet seasons. Exposure of jaguar faeces to sun or shade had no effect on FGM concentrations, despite significant differences in weather parameters. Analysis of faecal morphology proved unreliable in identifying faecal age. We conclude that the corticosterone RIA is suitable for assessing FGMs in free-ranging Belizean jaguars by surveying the same transects every 3–4 days in both seasons. The cortisol EIA can be used during the dry season, but there are possible shifts in metabolite immunoactivity in wet conditions. Assessment of adrenal activity in jaguars ranging areas of varying human disturbance is a timely application of this methodology in Belize.

## Abstract (precis)

We performed immunoassay and environmental validations to assess the effects of natural environmental exposure of jaguar faeces on glucocorticoid metabolites. Glucocorticoid concentrations were stable for 5 days with the corticosterone RIA. Scat aging by morphology was unreliable. Environmental validation is needed to determine field sample collection protocols for elusive species.

## Introduction

Non-invasive hormone monitoring (NHM) is widely used in a variety of wildlife species, but requires rigorous testing to ensure the validity of techniques employed to assess steroid metabolites in excreta, such as faeces (Möstl *et al.*, 2005; Palme, 2005; Touma and Palme, 2005). The applicability of this powerful tool has been demonstrated in several areas of animal research, such as ethology, reproductive physiology and animal health (Goymann *et al.*, 2001; Wielebnowski *et al.*, 2002; Brown, 2006; Franceschini *et al.*, 2008; Dorsey *et al.*, 2010). Most of the studies employing NHM, however, are performed in *ex situ* settings, such as zoos or research centres, where samples can be collected fresh and exposure to environmental conditions is limited. While a substantial amount of research using NHM has been conducted in free-ranging populations of primates, less has been done in other species of mammals, reptiles and amphibians (Schwarzenberger, 2007). The reasons for this disparity are attributed to limitations in observing fresh defaecations without eliciting adrenal activity by the presence of personnel performing sample collections, difficulties in assessing sample age and/or assigning collected samples to an individual, and instability of steroid hormone concentrations in faeces after defaecation (Möstl and Palme, 2002; Sheriff *et al.*, 2011). However, recent advances in the use of remote tracking have proved valuable for following individuals to collect scat without disturbance (Ganswindt *et al.*, 2010). Molecular techniques, such as DNA detection in animal excreta, are now employed to monitor populations non-invasively and assign individual identity or gender to animals from scat samples (Piggott *et al.*, 2006; Barja *et al.*, 2008). The remaining challenge is to ensure that faecal hormone metabolite concentrations are stable, within the time frame of collection, after deposited scat is exposed to natural environmental conditions.

The increased interest in employing NHM to aid wildlife conservation efforts has provided incentive to develop sampling methods that are robust in field conditions. For instance, primatologists have tested various sample storage conditions to avoid changes in hormone concentrations and facilitate transport of excreta and hormone extracts from the field to the laboratory (Khan *et al.*, 2002; Lynch *et al.*, 2003; Pappano *et al.*, 2010; Shutt *et al.*, 2012). Recent efforts in other mammals have targeted the design of ‘field-friendly’ techniques to increase the applicability of NHM to field endocrinology. Some of these methods include hormone extraction, sample storage and immunoassay of samples in field conditions (Freeman *et al.*, 2010; Santymire and Armstrong, 2010). It is widely accepted that hormonal metabolites in faeces are affected by factors such as temperature, humidity and ultraviolet rays, which can influence the presence of flora that alter hormone concentrations directly and indirectly through biotransformation processes (Macdonald *et al.*, 1983; Touma and Palme, 2005; Schwartz and Monfort, 2008). There is evidence that steroid hormones can increase or decrease in concentration after exposure to the environment (Washburn and Millspaugh, 2002; Abáigar *et al.*, 2010), although it is not always clear whether steroid hormone metabolites are genuinely changing in concentration or if there is a conversion of metabolites to forms that have higher or lower affinity to the antibody. The lack of this information for most species hampers our ability to use NHM in *in situ* wildlife studies (Kelly *et al.*, 2012). Another caveat arises in accurately ageing faecal samples exposed to environmental conditions. Most field-based studies determine faecal age through subjective judgement based on scat morphology, such as categorizing samples based on condition, colour, moisture level, odour strength, presence of mould and presence of invertebrates (e.g. Vynne *et al.*, 2012); however, these sample characteristics can vary dramatically based on environmental conditions other than simply scat age.

Most of the research using NHM in wild populations of elusive species has occurred in temperate environments, yet the effects of the environment on hormone metabolite concentrations have not been assessed directly in the majority of these studies. For instance, a survey of faecal glucocorticoid metabolites (FGMs) of Alaskan brown bears (*Ursus arctos horribilis*) conducted during the summer months in Katmai National Park, Alaska, considered 2 days as prudential time for hormone concentration stability (von der Ohe *et al.*, 2004). Other studies have been performed strictly during the snow-filled winter, thus avoiding any type of change in hormone metabolite concentrations, which are stable when frozen [e.g. elk (*Cervus elaphus*) and wolves (*Canis lupus*) in Yellowstone National Park, Creel *et al.*, 2002; capercaillie (*Tetrao urogallus*), Thiel *et al.*, 2008]. While there have been some attempts to evaluate the effects of environment on hormone concentration for NHM, in general the studies have been fairly limited (Fanson, 2009; Rehnus *et al.*, 2009; Abáigar *et al.*, 2010; Hulsman *et al.*, 2011).

The objectives of the present study were to validate a cortisol enzyme immunoassay (EIA), to perform biological validations of a cortisol EIA and a corticosterone radioimmunoassay (RIA) for measuring FGMs in jaguars (*Panthera onca*) and to conduct an environmental validation as a means of assessing the effects of a natural environment, with its climate variations (dry and wet seasons), on the stability of FGM post-defaecation in jaguar scat.

## Materials and methods

### Animals and sample collection

The jaguars (*Panthera onca*) included in this study were kept at the Belize Zoo, Belize, Central America. All individuals were wild-born adults (five males and four females, between ∼6 and 10 years of age), with the exception of one captive-born male (2 years of age). Most jaguars were not exposed to the public, except for two wild-born adults (exposure to the public for >2 years) and the captive-born male (exposure to the public for ∼1 year). Jaguars not exposed to the public were housed individually; each enclosure had two dens and a common yard, where individuals alternated yard use every 24 h, without having direct contact with each other. The diet consisted mainly of chicken carcasses and other animal products when available. Water was provided *ad libitum*.

### Study site and environment

The natural vegetation in Belize, a Neotropical country, is predominantly moist and wet broadleaf forest. In some areas, there are pine forests and more open pine savannahs. The climate follows bimodal tropical conditions separated by a cool transitional period. The dry season, from February to May, is characterized by higher temperatures ranging from 24 to 33°C and scarce rainfall (≤100 mm month^−1^). The wet season, from June to November, has slightly lower temperatures (18–28°C) and predominant rains, ∼60% of the annual precipitation, which on average fluctuates between 1500 and 2000 mm. The cool transition period occurs between November and January. Relative humidity oscillates around 80% year-round, with some fluctuations between the dry and wet seasons (Fuller and Wilson, 2002).

### Environmental validation

#### Study design

Fresh faecal samples (e.g. no older than 8 h) from all jaguars included in the study were collected from each individual at the Belize Zoo in both the wet and dry seasons over a 3 day period in each season (one or two samples per individual per season). Samples were placed in plastic bags and moved to the field site at the Tropical Education Centre (TEC) within 30 min, where they were exposed to two environmental conditions: shade (17.35707N, 088.54159W; 41 m elevation); and sun (17.35735N, 088.54121W; 39 m elevation). Faecal samples assigned to the shade treatment were placed under broadleaved trees that provided shade during daylight periods, whereas samples assigned to the sun treatment were placed under direct exposure to the sun. Collected faeces were thoroughly mixed before subsampling over time. A control (first sub-sample) was immediately frozen at −20°C to arrest degradation. Thereafter, daily sub-samples were collected from each scat and frozen over a 5 day period in both seasons. Morphological scat ageing was assessed using the following parameters: colour (dark brown, light brown, white) and moisture (scale: 1 = dry to 5 = very moist). Photographs of scats were also taken daily for later reference.

#### Weather variables

Temperature (in degrees Celsius), relative humidity (expressed as a percentage) and dew point (in degrees Celsius) were measured every 5 min with two data loggers (Lascar Electronics, EL-USB-2) placed at ground level in each set of each environmental conditions. A hand-held unit (Omega, HHUVA1) was used to measure ultraviolet (UV) rays (in watts per square metre) every 3 h at ground level during collection periods.

### Extraction of steroids from faeces

At the Smithsonian Conservation Biology Institute (SCBI), faeces were freeze-dried, homogenized and pulverized. Dry faeces were extracted (0.19 ± 0.01 g) in 5 ml of ethanol 90% (v/v) by boiling in a water bath (90–100°C) for 20 min and centrifuging at 500***g*** for 15 min. Supernatants were recovered and pellets resuspended in 5 ml of ethanol 90% (v/v), vortexed for 30 s and centrifuged again. Supernatants were combined and air-dried overnight. Extracts were resuspended in 1 ml of methanol and placed in an ultrasonic cleaner for 10 min. Extracts were diluted (1:1 v/v) with steroid buffer (0.1 m NaPO_4_ and 0.149 m NaCl, pH 7.0) and stored at −20°C until analysis.

### Glucocorticoid metabolite immunoassays

Two immunoassays validated for other carnivore species by Young *et al.* (2004) were tested to identify the best method for measuring FGMs in jaguar feces. The cortisol EIA employed a cortisol–horseradish peroxidase ligand and antiserum (no. R4866; C. J. Munro, University of California, Davis, CA, USA) and cortisol standards (hydrocortisone; Sigma-Aldrich Inc., St Louis, MO, USA). The polyclonal antiserum was raised in rabbits against cortisol-3-carboxymethyloxime linked to bovine serum albumin. The EIA was performed in 96-well microtitre plates (Nunc-Immuno™, Maxisorp™ Surface; Fisher Scientific, Pittsburgh, PA, USA) coated 14–18 h previously with cortisol antiserum (50 μl per well; diluted 1:20 000 in coating buffer; 0.05 m NaHCO_3_, pH 9.6). Faecal extracts, diluted 1:8 in steroid buffer (0.1 m NaPO_4_ and 0.149 m NaCl, pH 7.0), were analysed in duplicate. Cortisol standards (50 μl, range 3.9–1000 pg per well, diluted in assay buffer, 0.1 m NaPO_4_, 0.149 m NaCl and 0.1% bovine serum albumin, pH 7.0) and samples (50 μl) were combined with cortisol–horseradish peroxidase (50 μl, 1:8500 dilution in assay buffer). Following incubation at room temperature for 1 h, plates were washed five times before 100 μl substrate buffer [0.4 mm 2,2′-azino-di-(3-ethylbenzthiazoline sulfonic acid) diammonium salt, 1.6 mm H_2_O_2_ and 0.05 m citrate, pH 4.0] was added to each well. After incubation on a shaker for 10–15 min at room temperature, the absorbance was measured at 405 nm.

Faecal extracts were also analysed using a double-antibody ^125^I-labelled corticosterone RIA (MP Biomedicals, Orangeburg, NY, USA), previously validated for jaguar faeces (Conforti *et al.*, 2012), according to the manufacturer's instructions, except that all reagent volumes were halved. The polyclonal antiserum was raised in rabbits against corticosterone-3-carboxymethyloxime coupled to bovine serum albumin. Faecal extracts were diluted 1:250 in steroid diluent and analysed in duplicate. The inter-assay coefficient of variation (CV) of quality control samples run in each assay was <10% (cortisol EIA and corticosterone RIA: CV for high-dose control, 6.42 and 4.08%; CV for low-dose control, 9.65 and 8.04%; *n* * = * 14 and 3, respectively). The intra-assay CV was <10% for both immunoassays. Faecal glucocorticoid metabolite concentrations are expressed as nanograms per gram dry faecal matter (i.e. ng g^−1^).

### Immunoassay validation

Faecal extracts from fresh samples (i.e. controls for environmental validation) were used to conduct parallelism, exogenous corticosteroid accuracy recovery, high-performance liquid chromatography (HPLC) and a biological validation for each immunoassay.

#### Parallelism

A test for parallelism between the assay standards and a pool of faecal extract was performed for both the corticosterone and cortisol immunoassays. An equal amount of faecal extract from each fresh sample was pooled and diluted serially, 2-fold, in steroid buffer. Serial dilutions were analysed in duplicates for each assay.

#### Exogenous corticosteroid accuracy recovery

Exogenous corticosteroid accuracy recovery was tested by adding a known amount corticosteroid, according to the standard/original antigen of the immunoassay, to a pool of jaguar faecal steroid extracts. Serial dilutions of the spiked combinations were analysed in duplicate. The percentage steroid recovery was calculated based on two factors, the expected concentrations (known amount added) and the observed concentrations (observed concentration minus endogenous concentration of the pool), by incorporating them into the following formula:

$$\begin{array}{ll} & \text{Percentage steriod recovery}\\ & \;=(\text{Observed concentration/Expected concentration})\times 100. \end{array}$$

#### High-performance liquid chromatography

Reverse-phase HPLC was performed to analyse the immunoreactivity of a pool of faecal steroid extracts with the following known radioactive glucocorticoid tracers: ^3^H-cortisol, ^3^H-corticosterone and ^3^H-deoxycorticosterone. A pool of faecal steroid extracts, from three males and three females, was filtered, dried and spiked with radioactive hormone markers. Fifty-five microlitres of sample extract was separated on a Microsorb column (Reverse Phase Microsorb™ MV 100 C-18, 5 μm diameter particle size; Varian Inc., Woburn, MA, USA) using a gradient of 20–100% (methanol:water) over 80 min (1.0 ml min^−1^ flow rate, 1 ml fractions) as described by Young *et al.* (2004). The percentage of methanol remained constant at 20% for 5 min and increased linearly to 100% over the remaining time. A 100 μl aliquot of each fraction was used to analyse radioactivity (in counts per minute). The remaining 900 μl per fraction was air-dried overnight and reconstituted in 150 μl of distilled water for immunoreactivity analysis.

### Biological validation of immunoassays

Faeces from individuals that were assumed to undergo periods of high adrenal activity, i.e. a ‘stressful’ situation, were compared with faecal material from those that had not been exposed to a stressor. The Belize Zoo has a jaguar rehabilitation programme, which brings free-ranging, ‘problem jaguars’ into captivity to avoid the killing of jaguars by ranchers or villagers in retaliation for livestock losses. Free-ranging jaguars that are brought into captivity experience extreme adrenal challenges, such as transport, confinement, appetite loss and, potentially, self-injury. As jaguars acclimate to the new captive environment, their appetite increases, aggressive behaviours towards keeper staff decrease, and the jaguars spend less time hiding (personal communication from Sharon Matola, Director of the Belize Zoo). Jaguars are thought to take <1 year to acclimate, at least behaviourally, to captive conditions (personal communication from Sharon Matola). Furthermore, jaguars that spend >1 year in captivity show little or no aggressive behaviours and even affiliative expressions, such as body rubbing and vocalizations (e.g. gurgle and prusten) towards keeper staff (personal communication from Sharon Matola). Therefore, faecal samples were collected from each jaguar in the study, and individuals were grouped based on time spent in captivity as follows: <1 year (*n* = 2 males); between 1 and 4 years (*n* = 2 males and 2 females); >5 years (*n* = 1 male and 2 females); and captive born (*n* = 1 male). Four samples were collected per individual and averaged for statistical analysis. Jaguars recently brought into captivity (e.g. ≤1 year) were expected to have higher FGM concentrations than those individuals that were captive born or had been exposed to long-term captivity (e.g. >5 years).

### Statistical analysis

All analyses, except for the environmental validation assessment, were implemented in the statistical software JMP Pro 10 (version 10.0.2, 2012; SAS Institute Inc., Cary, NC, USA). All data were tested for normality before applying a statistical test. Normality was assessed with the Shapiro–Wilk goodness-of-fit test (α = 0.05). Data distributed in a non-normal fashion were logarithmically transformed. Multiple comparisons were performed when appropriate using the Tukey–Kramer HSD multiple comparisons test (α = 0.05). Biological validation was assessed by grouping FGM concentrations of jaguars by time spent in captivity using a one-way ANOVA. The test for parallelism was done with a multiple linear regression of logarithmically transformed concentration and the binding percentage of both the standard and serially diluted extracts; the least-squares means of the regression were linearly contrasted by standard and serially diluted samples, as suggested by Grotjan and Keel (1996). Expected and observed concentrations of the exogenous corticoid accuracy recovery analysis were tested using a simple linear regression for each immunoassay, the cortisol EIA and the corticosterone RIA. Environmental validation was assessed using a generalized linear mixed model constructed and run in the statistical software SAS (version 9.4, 2002–2012; SAS Institute Inc.), where FGM concentrations as repeated measures were a function of sex, season, environmental condition, time and their respective interactions. Scats of individual jaguars were assigned at random to seasons and treatments. Weather variables were condensed as daily averages. Condensed data were analysed in a nested model, where every weather variable was a function of the environmental condition (sun or shade) factor, which was nested in the season factor (wet and dry).

## Results

### Immunoassay validation

Both immunoassays showed parallel displacement between dilutions of steroid standard and faecal extracts, as follows: cortisol EIA, *t* = 1.47, *P* = 0.16; and corticosterone RIA, *t* = 0.54, *P* = 0.6. The cortisol EIA and the corticosterone RIA had a corticosteroid accuracy of recovery >90%; both immunoassays showed a strong positive relationship between the expected and observed concentrations (cortisol EIA, *P* < 0.0001, *r*^2^ = 0.9717; and corticosterone RIA, *P* < 0.0001, *r*^2^ = 0.9937).

**Figure 1:**
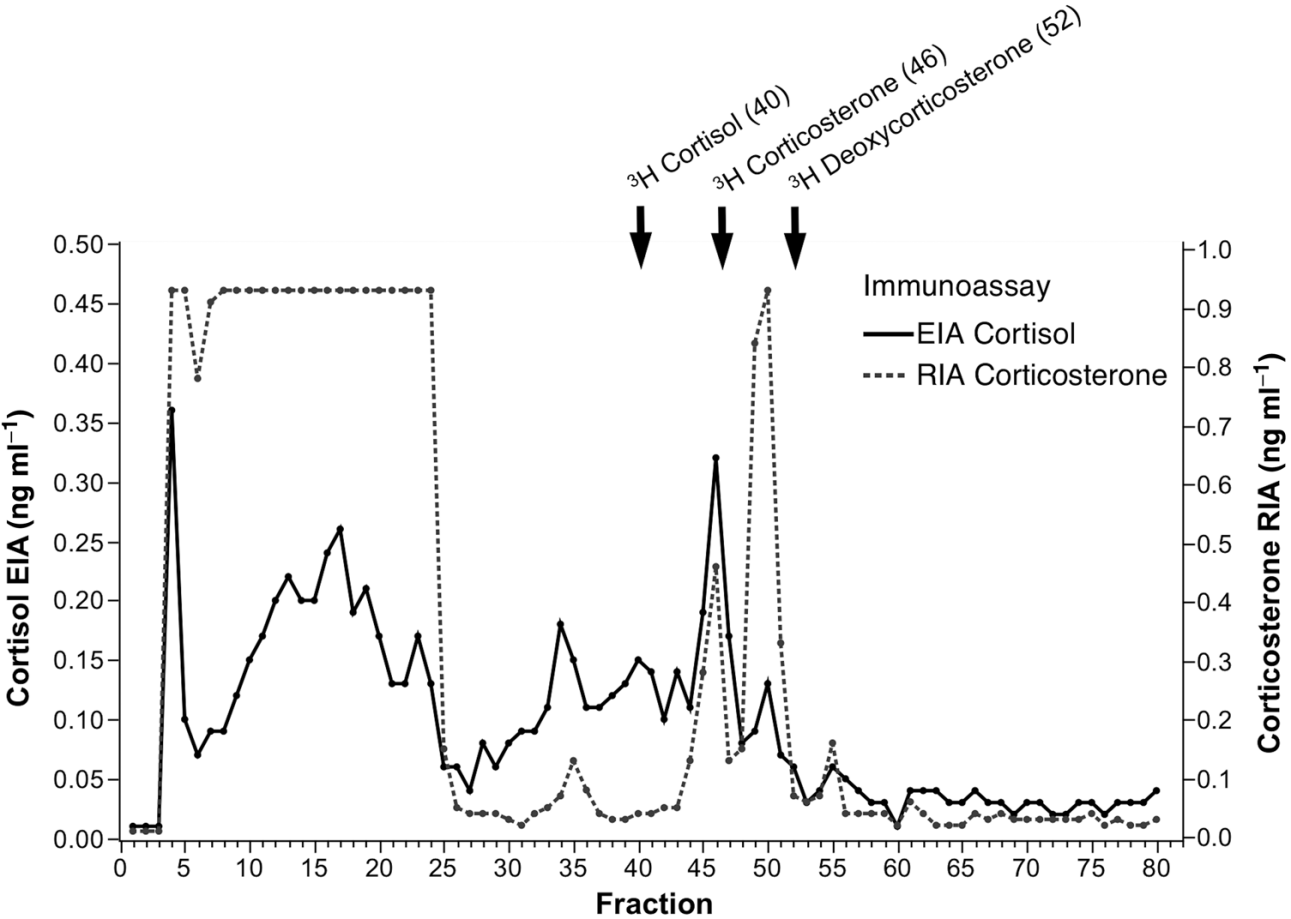
High-performance liquid chromatography profile of jaguar faecal glucocorticoi metabolites (FGMs) using two immunoassays, cortisol enzyme immunoassay (EIA; continuous line) and corticosterone radioimmunoassay (RIA; dotted line). The following three radiolabelled hormones were included: ^3^H-cortisol, ^3^H-corticosterone and ^3^H-deoxycorticosterone (arrows). Pooled faecal steroid extract was separated in a reverse-phase column (C18 μm diameter particle size) using a gradient of 20–100% methanol over 80 min (1.0 ml min^−1^ flow rate, 1 ml per fraction).

In the HPLC for the cortisol EIA, the majority of immunoactivity was observed in fractions 4 and 7–25, indicating the presence of highly polar glucocorticoid metabolites in jaguar faecal steroid extracts (Fig. 1). Another peak was observed at fraction 35. An immunoreactive peak at fraction 46 co-eluted with ^3^H-corticosterone. Similar immunoreactivity patterns were observed for the corticosterone RIA, with the exception of an additional peak at fractions 49–50. Again, most of the cross-reactivity was observed in fractions 4–25, indicating the presence of more polar glucocorticoid metabolites (Fig. 1).

**Figure 2:**
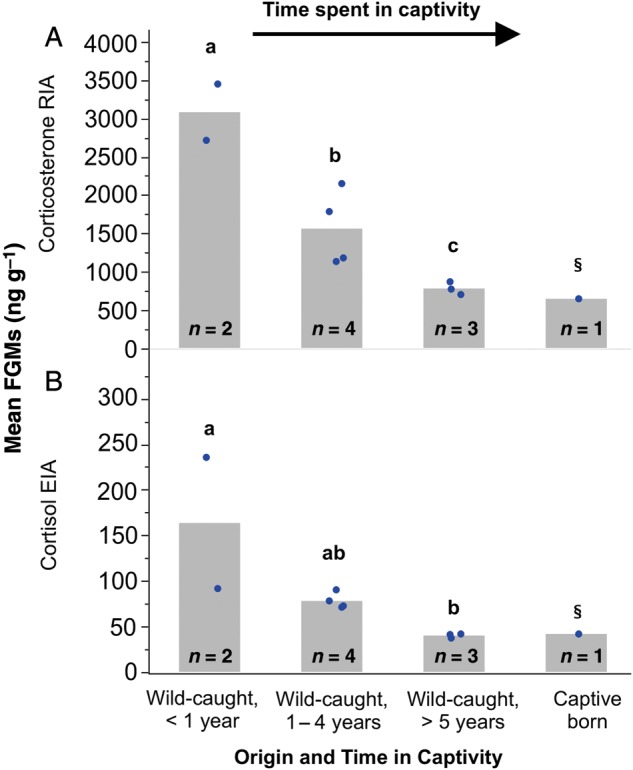
Biological validation of jaguar FGMs using two immunoassays, corticosterone RIA (A) and cortisol EIA (B). Mean FGM concentrations were measured in jaguars exposed to different durations in captivity after capture. Blue dots represent values for each individual. Bars with different letters are significantly different from each other; *n* is the number of individuals included in each category; §captive-born male not included in statistical test.

Faecal glucocorticoid metabolite concentrations of samples used for the biological validation were not normally distributed for either the cortisol EIA or the corticosterone RIA, but achieved normality after logarithmic transformations (cortisol EIA, *W* = 0.8855, *P* > 0.05; and corticosterone RIA, *W* = 0.9359, *P* > 0.05). Both immunoassays revealed that ‘problem jaguars’ recently captured from the wild and brought into captivity excreted 5-fold more FGMs than long-term captive jaguars (cortisol EIA, *F* = 12.79, *P* = 0.007, *r*^2^ = 0.81, power = 0.9901; and corticosterone RIA, *F* = 19.70, *P* = 0.0023, *r*^2^ = 0.868, power = 0.906) or a captive-born individual. Additionally, FGM concentrations declined significantly with increasing time that jaguars spent in captivity, <1, between 1 and 4 or >5 years (Fig. 2).

### Environmental validation

#### Weather variables

**Figure 3:**
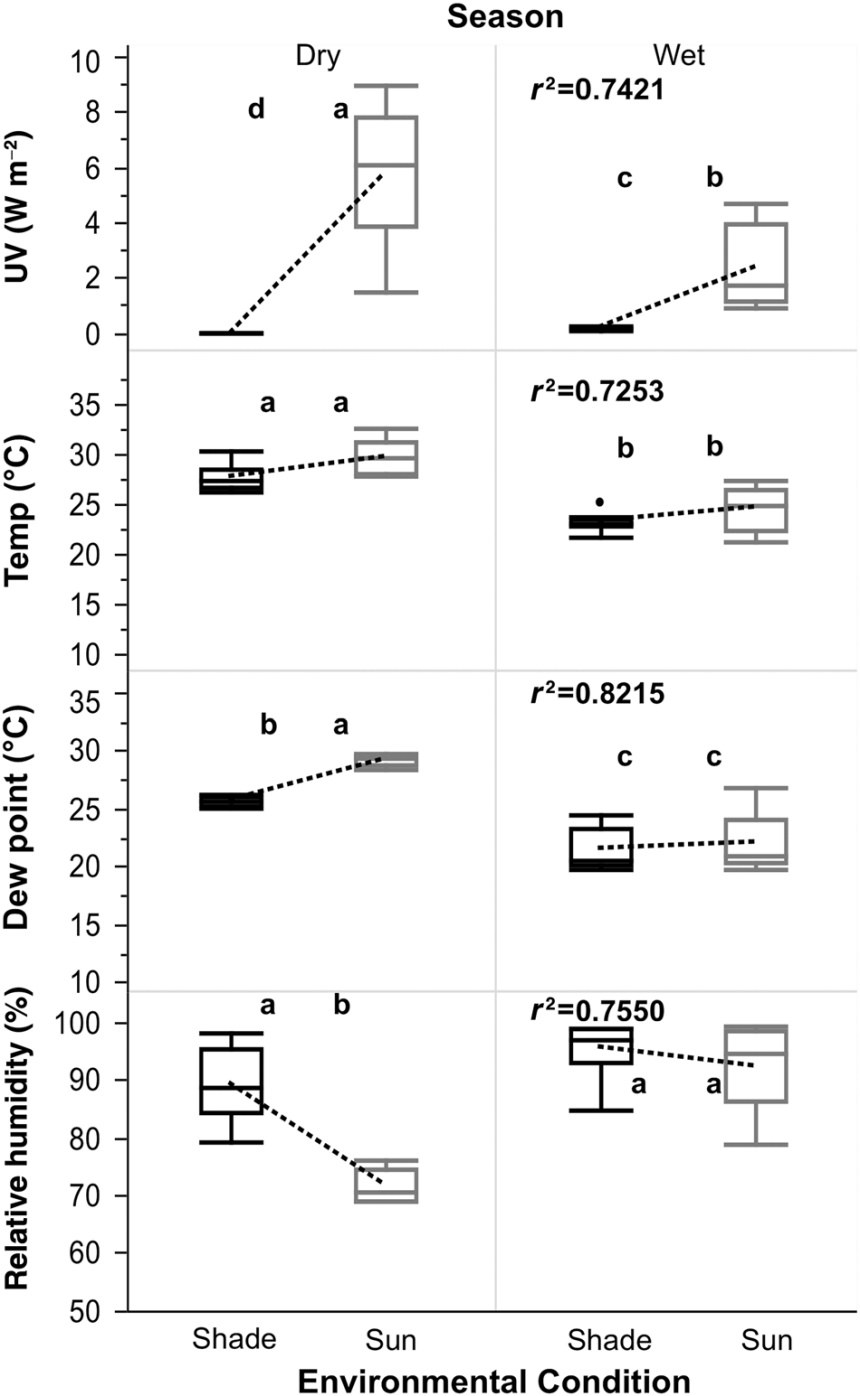
Climatic variables, summarized in box and whisker plots, associated with environmental validation of jaguar FGMs. The number of samples for each climatic variable in the dry and wet seasons was 40 and 64, respectively; *r*^2^ is the value of the square root of the significant model: weather variable = environmental condition (sun or shade) nested within a season (wet or dry) + error. Different letters denote significant differences. Dotted lines represent trends in environmental conditions within a season (temperature, d.f. = 3, *F* = 28.16, *P* < 0.0001; relative humidity, d.f. = 3, *F* = 32.88, *P* < 0.0001; dew point, d.f. = 3, *F* = 49.10, *P* < 0.0001; and UV, d.f. = 3, *F* = 30.69, *P* < 0.0001).

As shown in Fig. 3, all weather variables were significantly different between seasons (wet and dry) and environmental conditions (sun or shade).

#### Faecal glucocorticoid metabolite measures

**Figure 4:**
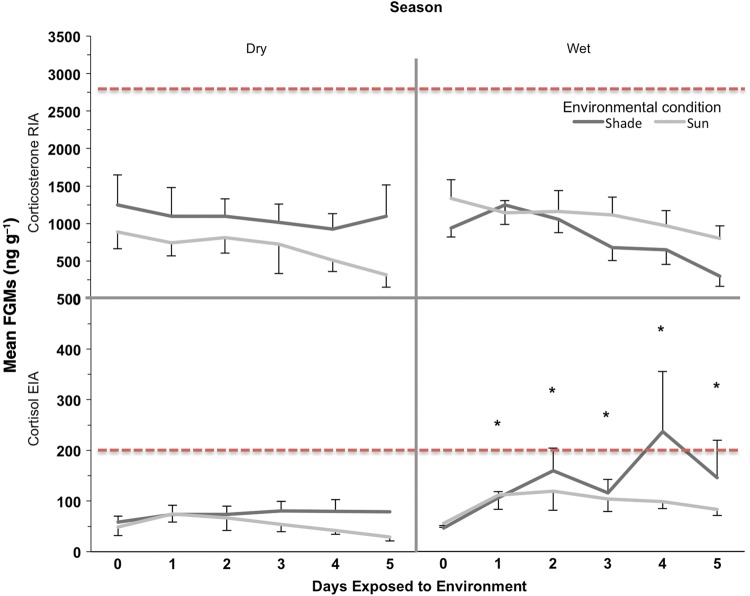
Faecal glucocorticoid metabolite concentrations assessed by two immunoassays, corticosterone RIA (top panel) and cortisol EIA (bottom panel) in jaguar faecal samples exposed for 5 days to two environmental conditions: sun (light line) and shade (dark line), in two seasons: wet and dry. Individual jaguars included in the dry season (shade *n* = 7, sun *n* = 9) and the wet season (shade *n* = 6, sun *n* = 6). Error bars represent 1 SEM. *Significant difference from control sample or day 0 of exposure. Horizontal red dashed lines represent the average FGM concentrations of individuals with high adrenal activity (i.e. <1 year captivity) from biological validation.

Faecal glucocorticoid metabolite concentrations achieved normality after logarithmic transformation for both assays (cortisol EIA, *W* = 0.8869, *P* < 0.05; and corticosterone RIA, *W* = 0.8971, *P* < 0.05). Statistical analysis indicated that FGMs, measured with the cortisol EIA, were more stable overall in the dry season than in the wet season (d.f. = 20, *F* = 5.27, *P* = 0.032). In contrast, no statistical difference between seasons was found in FGMs measured with the corticosterone RIA (d.f. = 20, *F* = 1.88, *P* = 0.18). Furthermore, time after collection was a highly significant factor contributing to stability in FGM concentrations for both assays (cortisol EIA, d.f. = 100, *F* = 6.17, *P* < 0.0001; and corticosterone RIA, d.f. = 100, *F* = 5.4, *P* = 0.0002). Faecal glucocorticoid metabolite concentrations measured with the cortisol EIA during the dry season were stable for 5 days (d.f. = 100, *t* = −0.25, adjusted *P* > 0.5), while stability of FGM concentrations during the wet season was <1 day (d.f. = 100, *t* = −5.27, adjusted *P* < 0.0001). Faecal glucocorticoid metabolite concentrations measured with the corticosterone RIA were stable for 5 days in both dry (d.f. = 100, *t* = 2.89, adjusted *P* = 0.16) and wet seasons (d.f. = 100, *t* = 3.20, adjusted *P* = 0.075). The influence of sex was not statistically significant on changes in FGM concentration stability over time for either immunoassay (cortisol EIA, d.f. = 100, *F* = 0.47, *P* = 0.796; and corticosterone RIA, d.f. = 100, *F* = 0.44, *P* = 0.817). Lastly, environmental conditions (i.e. samples exposed to sun or shade) did not have significant effects on the stability of FGM concentrations for either assay (cortisol EIA, d.f. = 24, *F* = 1.70, *P* = 0.204; and corticosterone RIA: d.f. = 24, *F* = 0.32, *P* = 0.575; Fig. 4).

#### Scat age assessment

**Figure 5:**
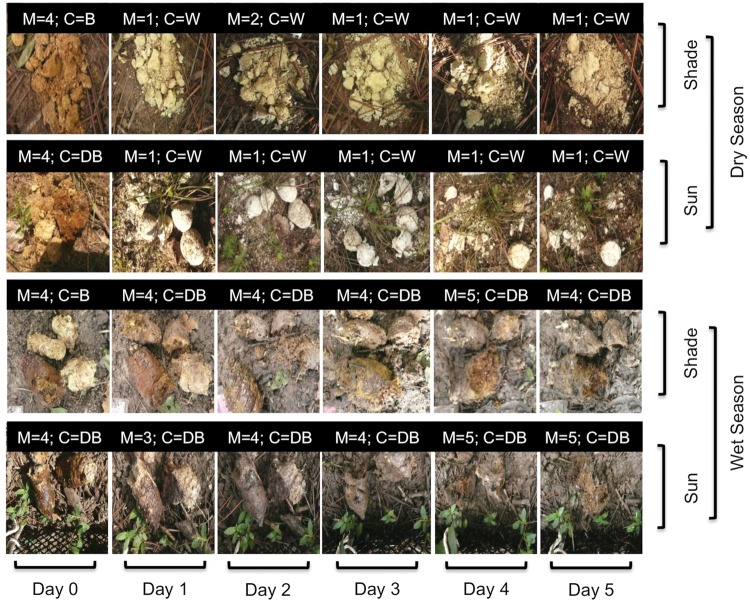
Morphology of jaguar scat during 5 days of exposure during the wet and dry seasons in two environmental conditions: sun and shade. An example of four scats photographed over time (row direction) is shown for every combination of environmental conditions. Morphological characteristic scores [M = moisture, from driest (1) to moistest (5); C = colour (DB, dark brown; B, brown; and W, white)] are presented in the black boxes.

We were unable to determine accurately the age of scat exposed to different environmental conditions. Moisture and, to a lesser degree, colour changed markedly after a scat was exposed to the environment. Scats gained moisture after rainfall, causing the colour to turn darker. In general, scat samples became dryer and whiter over time in the dry season, but no such trend was observed in the wet season (Fig. 5).

## Discussion

A cortisol EIA (R-4866 antibody) was validated to measure FGMs in jaguar scat. The results of the corticosterone RIA (MP Biomedicals) validation were congruent with a previous immunoassay validation performed by Conforti *et al.* (2012). Both immunoassays showed satisfactory parallel displacement and evidence of multiple glucocorticoid metabolites based on HPLC analysis, including an important amount of immunoactivity associated with more polar metabolites, similar to that observed in other felid species (i.e. cheetah, domestic cat, clouded leopard; Young *et al.*, 2004). Contrary to those species, jaguar extracts also contained immunoactive peaks that co-eluted with ^3^H-corticosterone, which is feasible for the RIA that uses a corticosterone antibody; however, the cortisol antibody cross-reacts only 0.7% with this glucocorticoid, so it is unlikely to be native steroid in that case. For this HPLC analysis, we used a pool of faecal extracts from male and female jaguars. Unfortunately, we were unable to make inferences regarding sex-related differences in HPLC profiles of FGMs. Other studies have shown that there are sex-related differences in FGMs (Touma *et al.*, 2003). Future studies could perform HPLC or other chromatographic techniques (e.g. gas chromatography coupled with mass spectrometry) to discern any potential differences in the array of FGMs between the sexes.

Biological validity of both assays was demonstrated by showing a significant difference in FGM among individuals in relationship to the time spent in captivity. As expected, individuals that had spent <1 year in captivity exhibited much higher adrenal activity than those with 1–4 or >5 years in captivity or a captive-born individual. These results are also consistent with behavioural differences among jaguars relative to time in captivity noted by the Belize Zoo director and staff (personal communication from Sharon Matola). The biological validity of the corticosterone RIA is further supported by the results of a previous study of captive jaguars that were challenged with exogenous adrenocorticotrophic hormone (Conforti *et al.*, 2012). Although different steroid extraction techniques were used [boiling (present study) vs. shaking (Conforti *et al.*, 2012)], the FGM concentrations assessed with the corticosterone RIA of the captive-born jaguar and the individuals that had spent <1 year in captivity at the Belize Zoo were similar to the baseline and the adrenocorticotrophic hormone-challenged jaguars, respectively, in the study by Conforti *et al.* (2012). Thus, based on laboratory and biological validations, both immunoassays appear to be effective at measuring FGM in fresh jaguar scat samples. Moreover, our sample sizes were too small to separate by sex in the biological validation; it still remains unclear whether jaguar males or females display different FGM profiles after challenging the hypothalamic–pituitary–adrenal axis.

A few investigators have assessed the change of faecal steroid metabolite concentrations over time, although the terminology differs, e.g. washing-out experiment (Rehnus *et al.*, 2009), effects of faecal age and seasonality (Abáigar *et al.*, 2010), field stability experiment (Fanson, 2009) and rate of FGM degradation (Hulsman *et al.*, 2011). Regardless of terminology, systematic experimental assessments of the effects of natural environmental conditions on hormone metabolites in excreta of wild animals are crucial to ensure methodological validity. In our environmental validation, the results differed somewhat for the two immunoassays. Faecal glucocorticoid metabolite concentrations did not differ statistically between seasons using the corticosterone RIA. In contrast, the cortisol EIA indicated that jaguar FGM immunoreactivity changes, possibly due to steroid degradation, at faster rates and with more variability during the wet season than during the dry season. Overall FGM concentrations also fluctuated more during the wet season for both immunoassays. However, a stronger effect was seen in the cortisol EIA, where FGM in older samples could mistakenly identify an individual as having higher adrenal activity. Both immunoassays showed that FGM concentrations changed over time when exposed to the environment; however, the effects were variable. Faecal glucocorticoid metabolite concentrations measured with the corticosterone RIA did not differ statistically after 5 days of exposure in either season. In contrast, FGM concentrations measured with the cortisol EIA were stable for <1 day of environmental exposure during the wet season. During the dry season, little change in FGM concentrations over time during was observed for both the cortisol EIA and the corticosterone RIA. Selection of the appropriate immunoassay, in this case, for measuring FGM in Belizean free-ranging jaguars, ultimately depends on the research question and goals. The corticosterone RIA is robust against the effect of climate seasonality, while the cortisol EIA should be used for scat field surveys only during the dry season. The contrasting outcomes from these immunoassays suggest the importance of evaluating more than one immunoassay in environmental validations. This practice could increase the chance of capturing or confirming relevant changes in steroid metabolite concentrations.

Even though sex differences in FGM concentrations have been shown to occur naturally in some felid species, such as Canada lynx (*Lynx canadensis*; Fanson *et al.*, 2012), our environmental validation showed no evidence of sex-related effects on the change of FGM concentrations of jaguar faecal samples exposed to the environment after defaecation.

Warmer and dryer weather appeared to minimize variation in FGM concentrations. Cooler and wetter weather possibly creates a more hospitable environment for micro-organisms with steroid biotransformation capacities, as previously suggested (Washburn and Millspaugh, 2002). Within season, vast differences in scat exposure to UV -rays among environmental conditions (e.g. 30 and 10 times higher UV intensity in sun vs. shade treatments in dry and wet seasons, respectively) did not appear to have an important effect on FGM concentrations. These results are perhaps explained by the low capacity of UV rays to penetrate solid structures, such as faeces (for example, Abdelrahman *et al.*, 1979). Therefore, the effects of UV-ray exposure on FGM degradation might be limited to the surface of the scat (i.e. depth of micrometres), whereas faecal steroids are widely distributed throughout the entire faecal sample.

Scat ageing by assessment of morphology in relationship to hormone stability was not successful because of high variation in colour and moisture changes after rain events. Additionally, scats turn white very quickly in dry conditions, which is likely to lead to overestimates of sample age. Furthermore, we observed that humidity drastically altered the morphology of faecal samples, making the ageing of scats in the field by morphology inaccurate. Instead, we recommend adjusting survey regimens to incorporate the results of environmental validations by resurveying an area at intervals to ensure stability in hormone concentrations in excreta as described below.

We suggest that those researchers interested in NHM sampling for jaguars in a tropical country, such as Belize, should conduct surveys in the dry season by using either the cortisol EIA or the corticosterone RIA, while surveys in the wet season should use only the corticosterone RIA. We found that scats cannot be aged accurately by morphology, but because FGM concentrations are stable for up to 5 days, sampling can involve an initial clearing of all scats from the target area and, to be conservative, resurveying every 3–4 days to ensure accuracy in hormone concentrations. A different sampling regimen could be adopted during the wet season, and if the cortisol EIA is used, resurveying would need to take place daily; however, strict caution in the interpretation of data should be taken due to the rapid change in FGM concentrations. In addition, daily survey may be impractical in field conditions.

Assessing the degree of stress in wild jaguars ranging across areas of varying human disturbance is a timely application of these methodologies. Belize has experienced a progressive increase in human activities such as hunting, housing developments, forest eradication and land conversion for agriculture, hence increasing levels of human–jaguar conflict. Our methods will ensure physiologically relevant FGM concentrations in faeces and could advance conservation physiology for jaguars by exploration of the linkage of habitat fragmentation and human–wildlife conflict to measures of adrenal activity in free-living jaguars.

## Funding

This work was supported by Virginia Tech and the Smithsonian Institution. The authors have not received any financial benefits from this publication.
